# Characterization of “Responder” in patients with Treatment-Resistant Schizophrenia (TRS) treated with a new antipsychotic added to their current antipsychotic monotherapy

**DOI:** 10.1192/j.eurpsy.2023.1330

**Published:** 2023-07-19

**Authors:** R. Anand, R. Hartman, A. Turolla, G. Chinellato

**Affiliations:** 1 R&D department, Anand Pharma Consulting AG, St. Moritz, Switzerland; 2 NeurWrite LLC, Morristown, United States; 3 R&D department; 4 Newron Pharmaceuticals SpA, Bresso, Italy

## Abstract

**Introduction:**

Numerous authors have proposed “responder” criteria for patients with schizophrenia treated with antipsychotic monotherapy (Leucht, S et al 2009; 438 7-14; Suzuki T et al, 2012; 197 1-6; Kane J et al 1988; 45 789-96). These suggest reductions greater than 30% on the PANSS total score, improvements of 1 category or more on the CGI-S, or CGI-C ratings of very much, much or minimally improved, as well as various permutations and combinations of the above. No study has met the responder definition of Kane et al in the last 30 years in monotherapy studies in TRS patients. However, a widely accepted definition of response in patients with TRS treated with a putative antipsychotic added to their background antipsychotic monotherapy, is not currently available, and more work is needed on this highly relevant topic (Suzuki, T et al 2011; 133 1-3).

**Objectives:**

Combining PANSS (30-item anchored scale), CGI-C and CGI-S (both 7-point Likert scales), three of the most accepted scales to evaluate patients with schizophrenia worldwide, we propose two different definitions of response in TRS population

**Methods:**

Study 014 was designed to evaluate the safety and preliminary evidence of efficacy of evenamide, a NCE added to an antipsychotic monotherapy, given orally at 3 fixed doses (7.5, 15 and 30 mg bid) in patients with TRS not adequately responding to a therapeutic dose of an AP. Assessment of efficacy was based on changes of the PANSS and CGI-S/C. We reviewed the efficacy data of the first 100 patients at various timepoints up to 30 weeks.

**Results:**

We assessed multiple definitions involving all the three measures (PANSS, CGI-S, and CGI-C) to determine one that would define a “responder” by categories that may be clinically meaningful. Review of the data indicated two definitions of responders based on the different combinations of the individual measures. “Full responder” was defined as PANSS total score improvement ≥ 20%; CGI-C at least much improved (i.e. 1,2); CGI-S at least one point improvement and reaching at least mildly ill (i.e. a score of at least 3 or less). “Partial responder” was defined as PANSS total score improvement ≥ 15%; CGI-C rated as any improvement (i.e. 1,2,3); CGI-S at least one point improvement. These two categories are alternatively true and patients not fulfilling the criteria for the above categories are considered as “non-responders”. Further descriptive analysis will be presented.

**Conclusions:**

These definitions may change the selection of compounds used as add-on therapy for TRS patients as well as the study designs to evaluate them.

**Disclosure of Interest:**

None Declared

